# Exploring Multidimensional and Within-Food Group Diversity for Diet Quality and Long-Term Health in High-Income Countries

**DOI:** 10.1016/j.advnut.2024.100278

**Published:** 2024-09-03

**Authors:** Anaëlle Bolo, Eric Verger, Hélène Fouillet, François Mariotti

**Affiliations:** 1 Université Paris-Saclay, AgroParisTech, INRAE, UMR PNCA, Palaiseau, France; 2 MoISA, Univ Montpellier, CIHEAM-IAMM, CIRAD, INRAE, Institut Agro, IRD, Montpellier, France

**Keywords:** nutritional adequacy, diet quality, healthy diets, dietary diversity, within-food group diversity

## Abstract

Dietary diversity is a crucial component of healthy eating patterns because it ensures nutritional adequacy. Yet, concerns have been raised about the potential risks of its increase, which may reflect excessive consumption of unhealthy foods and higher obesity or cardiometabolic risk, particularly in high-income countries. However, the links between dietary diversity and different health outcomes remain inconclusive because of methodological differences in assessing dietary diversity. Numerous studies, mostly cross-sectional, have assessed dietary diversity using different indicators usually based only on the number of foods or food groups consumed. In this perspective, we emphasize that dietary diversity is a multidimensional concept encompassing the number of foods in the diet (food coverage) but also their relative proportions (food evenness) and the nutritional dissimilarity of foods consumed over time (food complementarity). Consequently, a comprehensive assessment of dietary diversity reflecting all its dimensions, both between and within-food groups, is needed to determine the optimal level of complementarity between and within-food groups required to improve health and diet quality. Moreover, given the prevailing context of abundant highly processed and energy-dense foods in high-income countries, promoting dietary diversity should prioritize nutrient-dense food groups. Until recently, within-food group diversity has received limited attention in research and public health recommendations. Still, it may play a role in improving diet quality and long-term health. This perspective aims to clarify the concept of dietary diversity and suggest research avenues that should be explored to better understand its associations with nutritional adequacy and health among adults in high-income countries.

**Statement of Significance**

This perspective highlights the importance of considering all dimensions of dietary diversity rather than simply counting foods. Furthermore, we argue that dietary diversity between and within-food groups needs to be considered to better understand how to improve nutritional adequacy and health in high-income countries.

## Introduction

Dietary diversity is a key aspect of our diets because no single food can provide all nutrients. By combining several foods, dietary patterns emerge and provide total nutrient intake. Consequently, consumption of different food groups is needed to meet all nutrient requirements, meaning a minimum level of dietary diversity ensures nutritional adequacy (Text Box 1). Although highly diverse diets have been associated with greater nutritional adequacy among adults [[1], [2], [3], [4], [5]], they have also been associated, in high-income countries, with higher consumption of unhealthy, non-recommended foods (for example, ultra-processed foods, refined grains, and sugar-sweetened beverages) and higher energy intakes [6,7]. Specifically, the benefits from the association between dietary diversity and nutritional adequacy can be canceled out, and the overall health value of the diet may deteriorate because of an increased consumption of unhealthy foods. Not only is the consumption of different foods or food groups necessary, but these different foods or food groups should be the nutrient-rich ones to ensure a healthy diet. Moreover, concerns have been raised about the increasing incidence of diet-related diseases in developed countries [8]. Currently, results on the association between dietary diversity and health outcomes such as obesity, body adiposity, or non-communicable diseases are inconsistent [[9], [10], [11]], but it is still important to consider the benefits and risks of greater dietary diversity for adults.

Text Box 1 Nutritional adequacy: having sufficient intakes of nutrients to meet the nutrient reference values [22].

Moderation: limiting the intake of nutrients that are detrimental to health when consumed in excess (for example, saturated fatty acids, sodium, sugar) but also the intake of nutrients with a tolerable upper intake level [22].

Nutritional quality refers to both nutritional adequacy and moderation.

Alt-text: Text Box 1

Public health authorities have strongly encouraged dietary diversity. As early as the 1990s, some high-income countries included dietary diversity in their national dietary guidelines. They did this by focusing on the wise selection of food groups to ensure nutritional adequacy, avoid overconsumption of harmful nutrients, and prevent non-communicable diseases. In the United States, people were advised in 1992 to “eat a variety of foods to get the energy, protein, vitamins, minerals, and fiber [they] need for good health” [12], which has now become “enjoy different foods and beverages within each food group” in the most recent dietary guidelines [13]. Also in 1992, the first recommendation in the Australian dietary guidelines was to “enjoy a wide variety of nutritious foods” [14], which has now become “enjoy a wide variety of nutritious foods from [the] five food groups every day” [15]. The same recommendation has been followed in the guidelines of European countries, which include between 5 and 12 food groups, depending on the country [16].

However, the notion of dietary diversity, particularly that of “good” diversity in terms of nutritional adequacy and long-term health risk, is still not well defined in research and public health recommendations and is poorly understood by the general audience. In general, dietary guidelines only mention dietary diversity between food groups without defining it, and do not address food choices within food groups except in a few countries, albeit not very explicitly [13,17,18]. It is not clear whether a diet is considered diverse if it includes one food from each food group or if a diverse diet needs to have different foods within each food group. Thus, there is still a lack of understanding about the details of how to select foods in these groups and, in particular, whether and to what extent some food groups should be more diverse than others. Although consuming a diversity of foods from the recommended food groups is now recognized as a beneficial characteristic of a dietary pattern [5,19,20], we still need a better understanding of what dietary diversity is, develop better indicators to assess it, and define what a favorable diversity might be [21]. This perspective aims to clarify what dietary diversity encompasses, both in terms of concepts and assessment methods, and to suggest research avenues that should be explored to better understand the associations between dietary diversity—between and within-food groups—and nutritional adequacy and health among adults in high-income countries.

## What Is Dietary Diversity?

Previous work has highlighted the complexity of defining and conceptualizing dietary diversity [10,21,23]. To our knowledge, there is no single, consensual definition of dietary diversity, and often, different scores are used without any prior definition or conceptualization of what dietary diversity encompasses. Ruel [24] defined dietary diversity as “the number of different foods or food groups consumed over a given reference period”: this implies that there are multidimensional counting issues with its measurement. In the first place, the reference period has yet to be determined. We can assume that a longer reference period is better for a complete overview of an individual’s dietary diversity. However, it is technically difficult to record every single meal of an individual for a long and representative period. Therefore, the reference period rarely exceeds a few days for an exhaustive and precise assessment of dietary diversity. Several authors [23,25,26] have proposed a dietary diversity with 3 dimensions: count, dissimilarity, and evenness (Text Box 2).

Text Box 2 Dietary diversity includes count, evenness, and dissimilarity of foods or food groups.

Count: number of different foods or food groups consumed; easy to estimate and interpret [26]; does not capture relative proportions, types, or nutrient contents of foods or food groups consumed.

Dissimilarity: differences in nutrient composition between foods or food groups consumed; provides information on nutrient diversity and complementary food combinations; requires food composition data and quantitative indicators involving distance measures [23].

Evenness: distribution of foods or food groups consumed; assesses how evenly intake is distributed; requires quantitative indicators but there is no consensus on which indicator to use [23,27].

Alt-text: Text Box 2

Count refers to food coverage, that is, knowing how much of all available foods one eats. However, beyond simply counting the number of foods consumed, assessing their dissimilarity allows a better assessment of how they differ regarding nutrient content. Eating fruits, vegetables, and legumes seems less diverse than eating grains, vegetables, and fish from a nutrient content perspective. Also, eating apricots, peaches, and plums, all stone fruits, appears less diverse than eating apricots, apples, and strawberries. The dimension of dissimilarity is related to the need for complementarity between foods or food groups for better nutritional adequacy, because it is through food combinations that overall nutrient intake is achieved. This is the basis for the importance of diversity: the foods or food groups considered in the diet must be dissimilar enough to complement each other. An item that is different from others in terms of nutrient content will be of interest if it provides nutrients that are not found in other items already consumed in the diet, therefore contributing to avoid nutrient deficiencies in the diet.

Evenness (or proportion) is another important and less commonly considered dimension of dietary diversity. It refers to the distribution and balance between food groups within a diet or between foods within a food group, that is, their relative contributions in terms of mass or energy intake. For instance, when considering 2 food groups, the diet is less diverse if one largely dominates the other (for example, 80:20) than if both contribute equally (50:50) to the total intake.

It is clear that we lack a shared definition and validated indicators of dietary diversity encompassing the various dimensions mentioned above (food number, dissimilarity/complementarity, between- and within-group evenness). Furthermore, as a critical factor for nutritional adequacy, diversity must be a continuous feature of the diet, that is, it must be the hallmark of the “usual diet” because a diet that is only occasionally diverse is less likely to secure nutrient intakes over the long term. Therefore, to study diversity, the dietary assessment should follow the usual diet over a long period, with highly detailed consumption to accurately account for within-group diversity.

## How to Quantitatively Estimate Dietary Diversity?

Many studies have attempted to assess dietary diversity in different populations, and several scores have been developed to compare and rank the diets of different populations.

Most of these scores are simply an enumeration of some or all of the foods or food groups consumed in a given time period, depending on the study’s objectives. For example, one can count all the foods consumed as in the Food Variety Score [10,19,[28], [29], [30]] or count the recommended (healthy) foods separately from the non-recommended (unhealthy) foods, as did Gregory et al. [31] or Roberts et al. [32]. One can also count the number of species consumed, as a measure of dietary biodiversity [27]. In the Minimum Dietary Diversity for Women of Reproductive Age [33], the Household Dietary Diversity Score [34], or the food group variety [2,19,30], one counts the number of food groups among a pre-established list of groups. In the Dietary Diversity Score, the number of subgroups consumed is divided by the total number of subgroups within each of the 5 major food groups [35,36]. In the context of developing countries, this type of score, which is easy to calculate and interpret, is very useful as a proxy for food access at the household level and nutritional adequacy at the individual level [34]. However, these scores are not very relevant and informative in the context of high-income countries or some urban areas in lower-income countries, where food access is relatively easier and food supply is greater, with diets characterized by excessive intakes of certain unhealthy food groups (that is, red meat, ultra-processed foods, sugar-sweetened beverages) and nutrients (that is, saturated fatty acids, sugars, sodium).

In France, Jacquemot et al. [37] developed the ORCHID score specifically for older adults, but it can be adapted to other populations. It reflects healthy dietary diversity that is in accordance with the French dietary guidelines by giving higher weight to food groups whose consumption is recommended. Other scores were also created to assess healthy dietary diversity. In Germany, Drescher et al. [38] developed the Healthy Food Diversity index, which was later adapted to the context of the United States by Vadiveloo et al. [7] as the United States-Healthy Food Diversity index. More than a simple count of foods, these 2 scores are based on the Berry index (also known as the Gini-Simpson index), which represents the probability that 2 different foods randomly selected in a diet belong to 2 different food groups: the higher this probability, the more diverse the diet in the evenness dimension, as high probability indicates that foods tend to be equally distributed in the diet [39]. In addition, this probability is weighted by the health value assigned to each food (based on its place in the Dietary Guidelines) in the Healthy Food Diversity and United States-Healthy Food Diversity scores to give higher rates to diets with a more balanced distribution of healthy foods. These diversity scores have the advantage of considering a dimension other than the simple number/coverage of foods, namely their evenness/balance, and aim to measure healthy diversity by giving more weight to the healthy foods. However, these scores cannot be used to study the relationship between dietary diversity and diet healthiness because they confound both aspects. Instead, scores that only consider the number and proportion of foods in the diet, regardless of whether they are healthy, such as the Quantitative Index for Dietary Diversity [40], should be considered for assessment. In fact, diversity scores based on this consideration have also been developed in other disciplines, such as ecology, and are ready to be applied to human nutrition. Chaudhary et al. [41] and Remans et al. [42] used the Shannon entropy—an index developed initially in mathematics—at the population level to assess the diversity of national food systems. Di Maso et al. [43] developed the Nutritional Functional Diversity score, adapted from the field of ecology, to estimate the dimension of dissimilarity. Further application of such scores to assess dietary diversity at the individual level could be a perspective for research.

Even more than the evenness dimension, dissimilarity is rarely explicitly considered when assessing dietary diversity. We found only a few studies [25,26,44,45] that assessed dissimilarity, usually using the Jaccard distance, a simple measure originally used in ecology and applied in nutrition to calculate how 2 foods or food groups differ in their nutrient content [25]. In most cases, however, dissimilarity is not considered explicitly but is often implicit when the assessment integrates different food groups. This is because more dissimilarity/complementarity is assumed between food groups than between foods within the same group.

In terms of both conceptualization and assessment, dietary diversity appears to be multidimensional and complex, encompassing several levels such as number of foods, dissimilarity/complementarity, and evenness/balance, which together contribute in complex ways to nutritional adequacy and long-term health. The scores developed to date, which do not consider all the dimensions of dietary diversity, have been used to investigate the relationship between dietary diversity and nutritional quality without producing consistent results in high-income countries.

## Contrasted Associations between Dietary Diversity and Nutritional Adequacy or Health in High-Income Countries

Regarding the relationship between dietary diversity and nutritional adequacy, most studies conducted in high-income countries, as expected, have reported a positive association [[1], [2], [3], [4], [5]]. However, in such a context, increased dietary diversity could also lead to excessive intake of nutrients, such as saturated fatty acids, sugars, or sodium, that need to be limited. Concerns about moderation or excess have been addressed in only a few studies, with more contrasting results recently reviewed [10]. Using the Probability of Adequate Nutrient Intake scoring system, which integrates 2 sub-scores reflecting the extent to which a diet meets the minimum values (adequation) without exceeding the maximum values (moderation) for nutrients, Bianchi et al. [46] showed that dietary diversity was positively associated with nutritional adequacy but negatively with nutrient moderation. When considering the dietary level of food groups rather than the nutrient level, Vandevijvere et al. [2] also reported that both men and women with more diverse diets had better dietary adequacy and balance between adequacy and moderation but were more likely to have dietary excess. The generalizability of these findings remains limited because of large differences between studies in dietary data, time period considered, study design, statistical models, and dietary diversity scores used [21]. In addition, dietary diversity scores varied and rarely included more than one dimension of diversity, often only the number of foods or food groups. Indeed, sometimes, the whole diet did not fit the pre-established list of food groups included in the diversity score used, resulting in the exclusion of some foods and food groups from the analysis, with some studies including as few as 5 distinct groups [4,47,48]. For example, beverages, sugars, and high-fat foods are often omitted, although they are important in the context of high-income countries. Failure to include these food groups, which significantly contribute to the intake of nutrients to be limited, may bias the interpretation of results on the relationship between dietary diversity and nutritional adequacy.

The relationship between dietary diversity and health has also been a research question for years, with many health outcomes studied [10,49] and contrasting results. In addition, the physiologic mechanism by which dietary diversity might affect health remains unclear. However, over the past decade, a general relationship between dietary diversity and species diversity in the gut microbiota has been documented, reviving interest in dietary diversity in Western diets [50,51]. Regarding overweight/obesity, an American study [52] found that BMI was positively associated with dietary diversity. In contrast, a systematic review [9] found that body adiposity was not significantly associated with total dietary diversity but was negatively associated with the diversity in recommended foods, whereas no association between dietary diversity and obesity was found in another systematic review by Salehi-Abargouei et al. [11]. These discrepancies may be due to heterogeneity in the assessment of dietary diversity across studies and accordingly, in a more recent systematic scoping review, Verger et al. [10] found that the associations between dietary diversity and body composition varied across studies. Regarding cardiometabolic risk, the first report was by Kant et al. [53], who concluded that a less diverse diet, in that it lacked several of the 5 major food groups, was associated with an increased risk of cardiovascular disease. However, the data from Kant et al. [53] were old from people recruited in the 1970s. Using more recent data, Otto et al. [25] showed that dietary dissimilarity was positively associated with metabolic risk (the higher the dissimilarity, the higher the metabolic risk). In another systematic review, Mozaffari et al. [49] found that higher dietary diversity was associated with lower incidence of cancer- and cardiovascular diseases-mortality only in certain subgroups of individuals. A systematic review and meta-analysis conducted by Qorbani et al. [54] showed an inverse association between dietary diversity scores and levels of triglyceride but no association between dietary diversity scores and other cardiometabolic risk factors. For the same reasons as for nutritional adequacy (between-study differences in diversity scores, dietary data, and exclusion of foods), it is not possible to conclude about the importance of dietary diversity for long-term health, especially in a Western context of generally easy and abundant access to both healthy and unhealthy foods.

## What Degree of Dietary Diversity is Desirable in High-Income Countries?

Blanket statements about the importance of dietary diversity may encourage overconsumption of discretionary and energy-dense foods, highlighting the importance of specifying the type of groups in which diversity should be encouraged. Therefore, dietary diversity should not be separated from dietary guidelines that specify which food categories contribute to diet quality. This has already been argued by those who have advocated scores that combine healthiness and diversity [7], and by those who have shown that dietary quality (as measured by adherence to dietary guidelines) is actually much more important than overall dietary diversity for long-term health in high-income countries [6,25,55].

**FIGURE 1:**
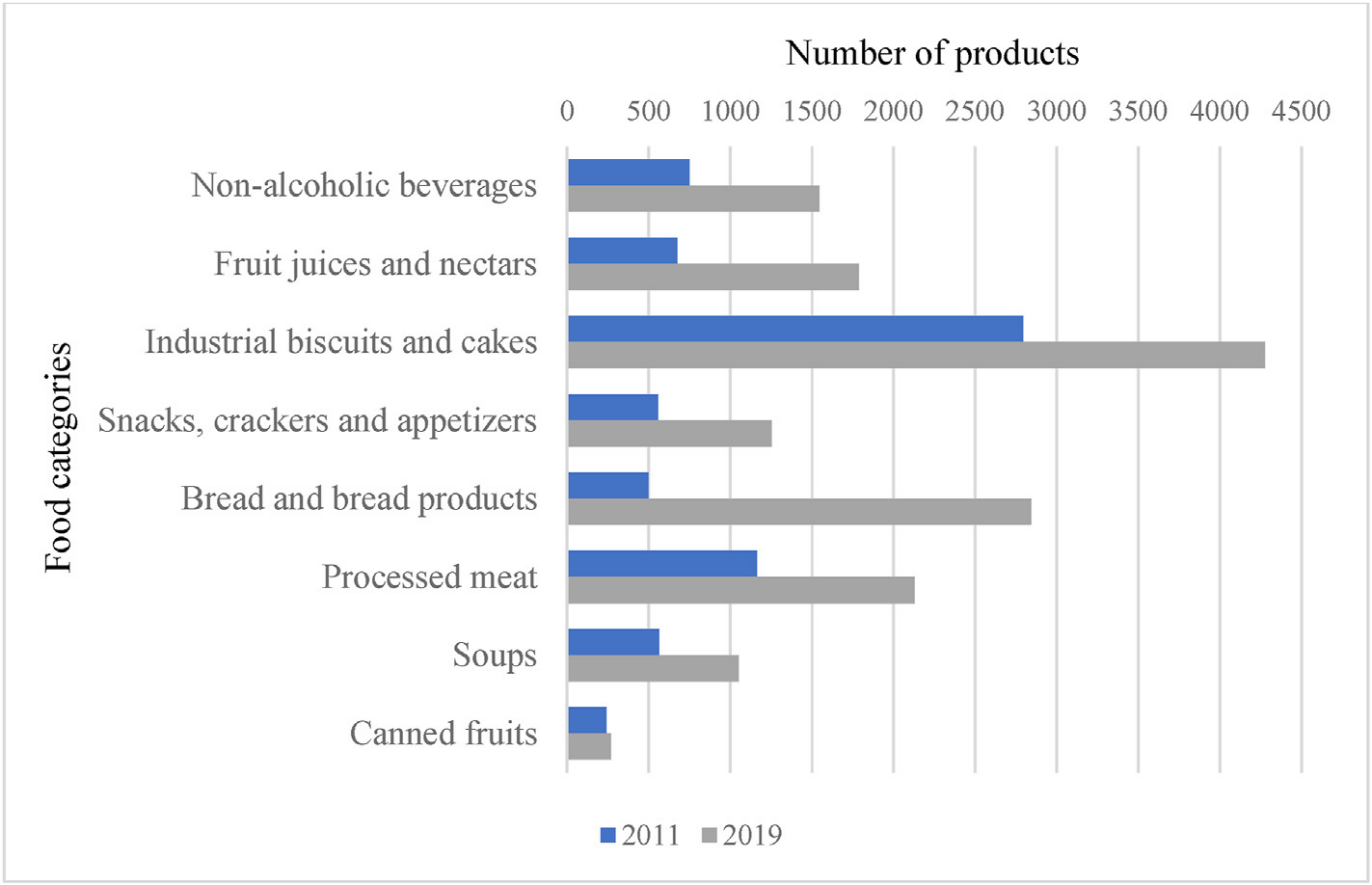
Number of products listed by the Food Quality Observatory in different food categories in 2011 and 2019.

Another consideration is how the food environment has undergone profound changes over the years, with more and more hyper-palatable foods high in fat, salt, and/or sugar, making them rewarding and difficult to stop eating [56,57]. Ultra-processed and energy-dense foods have also become increasingly present in the food supply of high-income countries as convenience foods [58]. In France, we found an example using data from the Food Quality Observatory [59,60], which lists the references of processed foods on the French market. In the categories that we considered to contain more ultra-processed foods, the number of registered references has increased significantly in recent years compared with, for example, canned fruits, a food category that we consider healthier although it includes less healthy versions such as fruits canned in syrup (Figure 1).

In the current context of abundance and high food availability, it has already been suggested that high overall dietary diversity is not necessary for health when living in a context of sufficient food supply. However, beneficial dietary diversity only concerns nutrient-dense foods [7]. In practice, this translates to increasing diversity by selecting nutrient-dense items within-food groups. On the basis of this, the United States adapted its 2015–2020 Dietary Guidelines by adding the statement “choose a variety of nutrient-dense foods across and within all food groups in recommended amounts” [61]. However, there are still unanswered questions, especially regarding which food groups and which specific dimension of diversity within them could be a lever for defining healthy diets.

**FIGURE 2:**
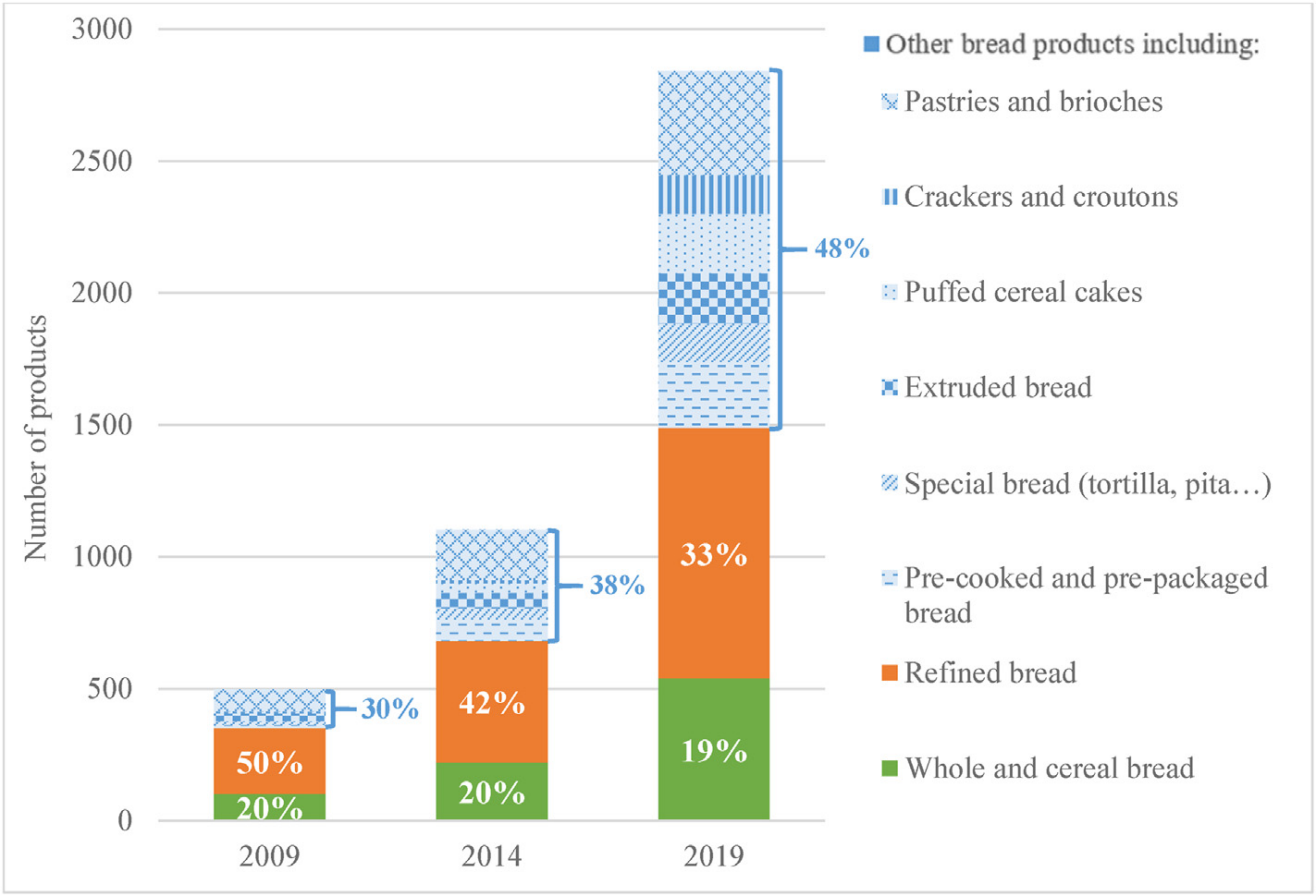
Evolution of the number of products in France in the category of bread products. Refined bread includes refined sandwich bread, toasted bread, buns, hamburger buns, and rusks. Whole and cereal bread includes whole and/or cereal sandwich bread, toasted bread, buns, hamburger buns, and rusks. Other bread products include pre-cooked and pre-packaged bread, special bread (tortilla, pita, etc.), extruded bread, puffed cereal cakes, crackers and croutons, pastries, and brioches. Data are from the Food Quality Observatory [60].

Another reality is that food supply trends can make increasing the diversity of nutrient-dense food groups such as whole grains, legumes, or fruits difficult. Although the number of food products in the marketplace has skyrocketed in recent years, this has not been uniform across and within-food groups. For example, using the French Food Quality Observatory longitudinal data, we found a larger range of products in subcategories such as crackers, chips, or candies. To investigate this finding further, we compared the changes in the number of products over time in 2 similar subcategories: refined bread products and bread products with whole grains or cereals (Figure 2 [60]). We found that the increase was twice as high in the former as in the latter. In proportion, the number of products identified as refined bread decreased from 50% of the bread category in 2009 to 33% in 2019. However, this decrease occurred in favor of other bread products, of which the proportion increased over the decade, but not in favor of whole and cereal bread, which has consistently represented around 20% of bread products.

## Is Within-Food Group Dietary Diversity the Key to Explaining the Relationship between Diversity and Nutritional Quality and Health?

We have yet to understand the associations between dietary diversity within-food group and nutritional quality or health. Although there is still insufficient research on this topic, findings from a few studies have been reported. In a study conducted in Belgium, Vandevijvere et al. [2], considering the dimension of count, showed that higher diversity within specific food groups was associated with better adherence to dietary guidelines. They found that dietary diversity within dairy or spreadable fats was positively associated with dietary adequacy and balance (adequate intake avoiding excess). In contrast, dietary diversity within energy-dense, nutrient-poor foods was negatively associated with balance. In another study, McCrory et al. [62] assessed dietary diversity by the percentage of food types within each food group regardless of frequency, so only the count dimension was considered, not the evenness dimension. They examined the relationship between dietary diversity within-food groups and energy intake or body fat and found that diversity in vegetables was negatively associated with body fat. In contrast, diversity in sweets, snacks, condiments, entrées, and carbohydrates group was positively associated. Still considering the dimension of count, Bhupathiraju and Tucker [63] found that diversity within fruits and vegetables, but not intake, was associated with reduced inflammation in Puerto Rican adults. In addition, Jeurnink et al. [64] showed that diversity in fruits and vegetables may decrease the risk of esophageal cancer, whereas Conrad et al. [65] found that variety in vegetables was not associated with the risk of mortality from all causes, cardiovascular disease, and coronary artery disease but that quantity consumed was. Considering the evenness dimension of dietary diversity, Sadohara et al. [66] found that higher diversity within the nuts, seeds, and legumes food group was associated with lower waist circumference.

Taken together, these findings call for further research to better investigate the effects of within-food group diversity to decipher the relationship between dietary diversity, nutritional quality and health. Indeed, they suggest that emphasizing greater diversity within the healthy and nutrient-dense food groups over other groups should translate into a greater likelihood of a healthy and nutritionally adequate diet. Indeed, variations in nutrient concentrations exist not only among foods but also among different varieties or species within a single food group [67]. Combining several foods or food groups wisely may create a diverse combination that provides overall nutrient intake and improves nutritional quality.

In particular, it would be interesting to investigate whether and to what extent diversity within-food groups could exert a lever effect on nutritional quality and health, apart from the effects of their consumption levels. To date, very few authors have studied diversity separately from quantity. For example, in the studies of Jeurnink et al. [64][] and Leenders et al. [68], the association between the count of fruits and vegetables and the risk of cancer was adjusted for the quantity consumed. We hypothesize that more diversity within a healthy food group, as well as implying a higher consumption of that group, may favor better nutritional adequacy in the diet. Such a positive effect of intra-category diversity might then be expected to vary according to the food category and its nutritional characteristics, such as nutrient homogeneity, complementary nutrient profiles, and the specific contribution of that food category to nutrient intake in the diet. Moreover, intra-category dietary diversity may also promote increased intake of other beneficial compounds, such as polyphenols, which are increasingly recognized as beneficial to health. Another benefit of within-food group diversity could be a reduced chemical risk because diversifying foods could also mean diversifying the types of possible contaminants and thus reducing exposure to each substance [69]. It could be the same with some plant foods that contain a variety of natural toxins that can be harmful to human health [70]. In terms of dimensionality, we also hypothesize that the effect of within-food group diversity could change depending on the dimension considered, particularly evenness or dissimilarity.

## Conclusions and Research Perspectives

An important and unresolved question is which dimension of dietary diversity, and in which food group, can be an effective lever for improving diet quality and health in high-income countries. To better understand the relationship between dietary diversity and diet quality and health in the context of sufficient food supply in high-income countries, this perspective revealed that we need to address the problem of assessing dietary diversity by using suitable data and comprehensive indicators for all dimensions. In addition, it is essential to consider dietary diversity within-food group to improve research findings on associations between diversity, nutritional quality, and health and to elaborate adequate recommendations. However, studying dietary diversity within-food groups requires a food classification system. Indeed, how foods are grouped can influence the association between dietary diversity and diet quality or health, but the food grouping is often different across studies. There are several ways to constitute food groups that can be based on nutritional content, protein source, food processing, cooking level, or physical state, to name a few. How foods are grouped may also depend on the objective of the studies. Ideally, to study dietary diversity and its link with nutritional quality, this grouping must reconcile homogeneity at a dietary practical level (proximity of use) with some degree of nutritional homogeneity within-food groups (proximity of content).

A diverse diet should also be an optimal combination of food groups and foods that limit exposure to harmful nutrients/substances. This diverse combination of foods could also greatly contribute to reducing environmental impact and financial cost, thus covering all aspects of a sustainable diet. Thus, dietary diversity, although a complex concept, has significant implications for public health and the sustainability of food systems.

## Author contributions

The authors’ responsibilities were as follows – FM, HF, EV, AB: designed research; AB: wrote the article with contributions from HF, FM, EV; AB: had primary responsibility for the final content; all authors: reviewed and commented on subsequent drafts of the manuscript; and all authors: read and approved the final manuscript.

## Conflict of interest

The authors declare that they have no known competing financial interests or personal relationships that could have appeared to influence the work reported in this article.

## Funding

The authors reported no funding received for this study.
